# ZBTB7A-mediated regulation of astrocytic glycolysis in neurodegenerative diseases: insights from literature review and bioinformatics prediction

**DOI:** 10.3389/fnagi.2026.1852019

**Published:** 2026-06-24

**Authors:** Ping Ye, Zhen Li, Xingang Cui, Hong Wu, Hong Jiang

**Affiliations:** 1 School of Laboratory Medicine, Nanchang Medical College, Nanchang, Jiangxi, China; 2 Key Laboratory of Environmental Stress and Chronic Disease Control and Prevention, Ministry of Education, China Medical University, Shenyang, Liaoning, China; 3 School of Public Health, China Medical University, Shenyang, Liaoning, China; 4 Nanchang Pujian Industrial Co., Ltd., Nanchang, Jiangxi, China; 5 School of Public Health, Mudanjiang Medical University, Mudanjiang, Heilongjiang, China; 6 Key Laboratory of Liaoning Province on Toxic and Biological Effects of Arsenic, Shenyang, Liaoning, China

**Keywords:** astrocyte-neuronal lactate shuttle, glycolysis, metabolic, neurodegenerative diseases, ZBTB7A

## Abstract

The incidence of neurodegenerative diseases, including Alzheimer’s disease (AD), continues to increase with the extension of human lifespan. However, their pathogenesis remains incompletely understood. Altered energy metabolism, particularly glucose metabolism involving glycolysis and oxidative phosphorylation, is widely recognized as an early pathological feature of neurodegenerative diseases. Astrocytes, the most numerous and widely distributed functional cells in the central nervous system (CNS), support neuronal energy demands through the astrocyte–neuronal lactate shuttle (ANLS). Glycolysis is a major pathway of astrocyte energy metabolism, and enhanced astrocytic glucose uptake and glycolytic flux may help attenuate the progression of neurodegenerative diseases such as AD. Zinc Finger and BTB Domain Containing 7A (ZBTB7A) is a POZ/BTB and Krüppel (POK) family transcription factor that has been implicated in the regulation of metabolic genes, including glycolysis-related genes, in several cellular contexts. However, its role in astrocyte glycolytic regulation under neurodegenerative conditions remains unclear. In this review, we summarize current knowledge of ZBTB7A biology, astrocyte glycolysis, and glial metabolic dysfunction in neurodegenerative diseases, and integrate published evidence with bioinformatics-based transcription factor binding prediction. Our analysis identified putative ZBTB7A-binding motifs in promoter regions of genes involved in glucose uptake, glycolytic flux, lactate production, and lactate transport. These findings suggest a potential association between ZBTB7A and the astrocytic glycolytic/lactate metabolic network. Therefore, this review provides a conceptual basis for future studies on ZBTB7A-associated transcriptional regulation in astrocyte metabolic remodeling and its potential relevance to neurodegenerative diseases.

## Highlights

- ZBTB7A emerges as a key regulator of astrocytic glycolysis.
- ZBTB7A links astrocyte metabolic remodeling to neuronal dysfunction.
- Targeting ZBTB7A-mediated astrocytic glycolysis may offer therapeutic opportunities for neurodegenerative diseases.

## 1 Introduction

The incidence of neurodegenerative diseases is increasing annually against the backdrop of an aging population and a changing living environment (Erkkinen et al., 2018; Zhang et al., 2021; Andronie-Cioara et al., 2023). Globally, the prevalence of neurodegenerative diseases in people over 65 years of age ranges from 4 to 8% in developed countries and is higher in developing countries (Erkkinen et al., 2018). The prevalence rate increases significantly with age, doubling with every 5-year increase in age after 65 years and reaching 20–30% after 85 years of age (Erkkinen et al., 2018). Patients with Alzheimer’s disease (AD) have irreversible cognitive decline (Twarowski and Herbet, 2023), patients with Parkinson’s disease (PD) have tremor and muscle tone (Chen et al., 2020), and patients with Huntington’s disease (HD) have chorea-like involuntary movements (Martí-Martínez and Valor, 2022). The onset of the clinical manifestations of these neurodegenerative diseases not only deprives individuals of their ability to remember, feel, think or make decisions but also places a heavy burden on families, health care and social systems.

The brain is highly dependent on the uptake of glucose for intelligent activities such as thinking and judgment, and energy metabolism is very active (Magistretti and Allaman, 2015; Watts et al., 2018). Neurons, as the most significant energy-consuming cell type in the human body, show a high sensitivity to any subtle abnormal changes in energy metabolism (Itoh et al., 2003; Bouzier-Sore et al., 2006; Boumezbeur et al., 2010). And the proposed astrocyte-neuron lactate shuttle theory (ANLS) (Pellerin and Magistretti, 1994) well explains the problem of how neurons can rapidly acquire energy under high activity state. That is, astrocytes take up glucose from the bloodstream and generate lactate via the glycolytic pathway, which provides an energy substrate for neurons (Miyamoto et al., 2019; Bonvento and Bolaños, 2021). Glycolysis is a series of consecutive and interrelated enzymatic reactions, comprising a total of 10 consecutive steps, all catalyzed by corresponding enzymes to ensure efficient and orderly energy supply (Fernie et al., 2004; Li et al., 2023). Defects in central nervous system (CNS) energy metabolism that are strongly associated with synaptic dysfunction have been observed in the early stages of AD (Finsterwald et al., 2021; Minhas et al., 2024). Among them, decreased flux of glycolysis, a metabolic pathway, is strongly associated with β-amyloid (Aβ) deposition, tau protein hyperphosphorylation, and apoptosis (Butterfield and Halliwell, 2019; Finsterwald et al., 2021; Zheng et al., 2021).

Zinc Finger and BTB Domain Containing 7A (ZBTB7A) is a member of the POK transcription factor family (Lunardi et al., 2013). Its C-terminal zinc finger structure specifically binds to the DNA promoter regions of target genes and then recruits other regulatory factors through its N-terminal POZ structure to synergistically regulate the expression of target genes (Jeong et al., 2023). Studies have shown that ZBTB7A acts as a transcriptional repressor of genes encoding key glycolytic components, directly binding promoters of glucose transporter protein 3(GLUT-3), platelet-specific platelet-type phosphofructokinase (PFKP), and pyruvate kinase (PKM) to inhibit their transcription (Liu et al., 2014); immunohistochemical staining also revealed high ZBTB7A expression in mouse orbitofrontal cortex astrocytes (Fulton et al., 2023). Although investigations specifically addressing ZBTB7A regulation of astrocyte glycolysis remain limited, the transcriptional role of ZBTB7A together with the central importance of astrocyte glycolysis in CNS energy metabolism and neurodegeneration motivate in-depth studies. Therefore, clarifying the regulatory relationship of ZBTB7A within the astrocyte glycolysis network and elucidating its mechanisms in neurodegenerative diseases is expected to provide new targets and ideas for treatment.

## 2 Overview of ZBTB7A

### 2.1 Fundamental structure of ZBTB7A

**FIGURE 1:**
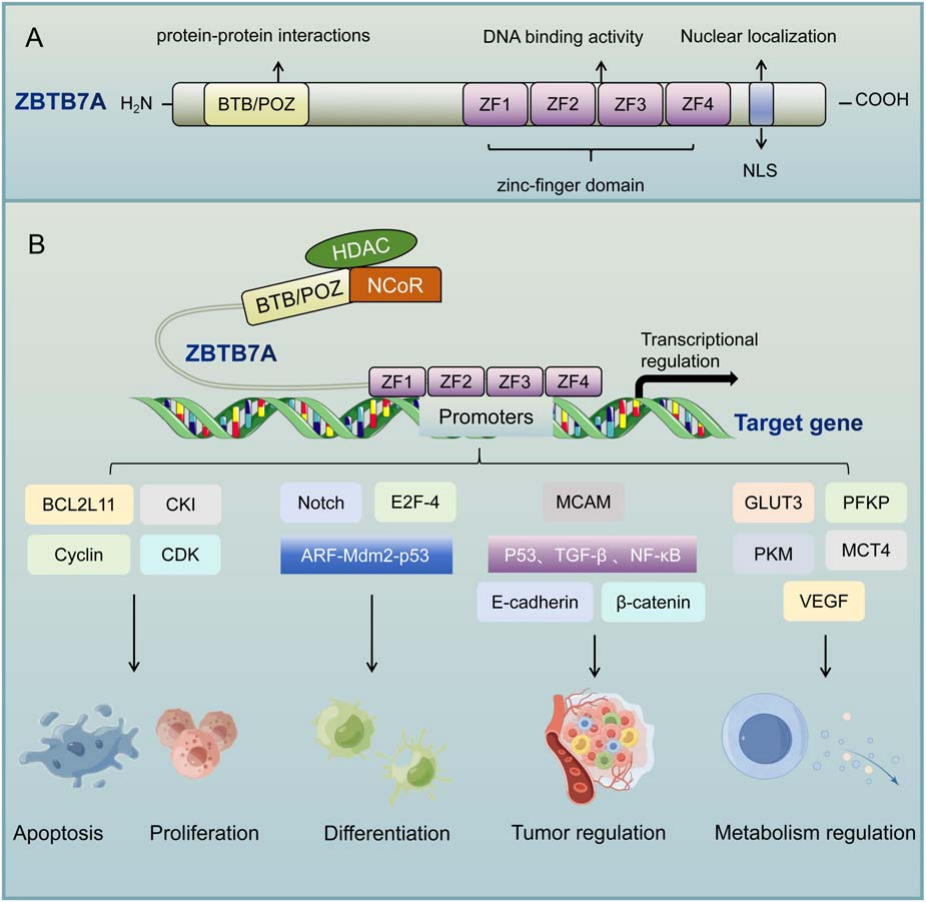
**(A)** The fundamental structures of ZBTB7A. The ZBTB7A protein consists of 584 amino acids and has a molecular weight of 86 kDa. The N-terminus of the ZBTB7A protein contains a POZ/BTB structural domain consisting of approximately 110 amino acid residues, which is responsible for mediating protein-protein interactions; the C-terminus of ZBTB7A contains four Krüppel-type zinc finger structures characterized by two pairs of conserved cysteine and histidine residues for the coordination of a Zn^2+^ Each zinc finger structural domain consists of approximately 30 amino acid residues, and the zinc finger structural domains confer on ZBTB7A the ability to bind to specific DNA sequences; a NLS is present in the C-terminal position 498 to the 502 amino acid residues at the C-terminus an NLS is present. **(B)** Overview of biological functions of ZBTB7A. The function of ZBTB7A proteins in a variety of biological processes in cells is mainly dependent on their transcriptional regulation. The zinc finger structural domain in ZBTB7A protein recognizes and binds to specific DNA sequences in the promoter region of target genes, and achieves targeted activation or inhibition of specific genes through non-covalent bonding such as hydrogen bonding and van der Waals forces. Once bound to the target gene promoter, the BTB structural domain of ZBTB7A can mediate interactions with other transcription factors, transcriptional coactivators or repressors to form transcriptional regulatory complexes. ZBTB7A proteins, as transcription factors, play a wide range of critical roles in organisms, involving involvement in cell proliferation and differentiation, tumor regulation, and metabolic regulation.

The human *ZBTB7A* gene is located on chromosome 19 (19p13.3) and encodes the protein ZBTB7A, also known as Pokémon, LRF, or FBI-1 (Lunardi et al., 2013). The ZBTB7A protein consists of 584 amino acids, has a molecular weight of 86 kDa, and is a member of the POZ/BTB and Krüppel (POK) family of transcription factors (Zhou et al., 2024). The structure of the ZBTB7A protein is characterized by a POZ/BTB structural domain at the N-terminus and four Krüppel-type zinc finger structures at the C-terminus (DiCiaccio et al., 2024). The POZ/BTB structural domain, among other domains, consists of approximately 110 amino acid residues. This structural domain is responsible for mediating protein–protein interactions, promoting the formation of homo or heterodimers of ZBTB7A and recruiting coregulators, such as histone deacetylase (HDAC) and nuclear receptor corepressor (NCoR), to collaborate in transcriptional regulatory processes (DiCiaccio et al., 2024). Zinc finger structural domains are characterized by two pairs of conserved cysteine and histidine residues that are used to coordinate Zn^2+^, thus maintaining a stable conformation of the zinc finger structural domain. Each zinc finger structural domain consists of approximately 30 amino acid residues, and the zinc finger structural domains confer on ZBTB7A the ability to bind to specific DNA sequences, enabling it to act as a transcription factor and precisely regulate the transcription of target genes (Gupta et al., 2020). In addition, there is a nuclear localization signal (NLS) at amino acid residues 498–502 in the C-terminus, which regulates the subcellular localization of the ZBTB7A protein in the nucleus (Gupta et al., 2020). The fundamental structure and transcriptional regulation process of ZBTB7A is shown in Figures 1A,B.

### 2.2 Biological functions of ZBTB7A

The function of the ZBTB7A protein in various biological processes in the cell is dependent mainly on its transcriptional regulation. The zinc finger domain of the ZBTB7A protein recognizes and binds to specific DNA sequences in the promoter regions of target genes, and through non-covalent bonding, such as hydrogen bonding and van der Waals forces, it can achieve targeted activation or repression of specific genes (Gupta et al., 2020). Once bound to the promoter of the target gene, the BTB domain of ZBTB7A can mediate interactions with other transcription factors, transcriptional coactivators or repressors to form a transcriptional regulatory complex. For example, histone acetyltransferases (HATs) are recruited to loosen the chromatin structure and promote the binding of RNA polymerase to the promoter to increase gene transcription; alternatively, HDACs are recruited to condense chromatin and repress gene transcription (Wang et al., 2020). Therefore, ZBTB7A proteins play extensive and critical roles as transcription factors in organisms and are involved in cell proliferation and differentiation, tumor regulation, metabolic regulation, and related processes (Chondrou et al., 2020; Yang et al., 2021; Ren et al., 2023). The biological functions of ZBTB7A is shown in Figure 1B.

#### 2.2.1 Cell proliferation and differentiation

ZBTB7A is activated by the erythrocyte-specific transcriptional regulator GATA1, which binds directly to the promoter region of the proapoptotic factor BCL2L11, negatively regulating its transcription and effectively inhibiting apoptosis (Xu et al., 2022). SAM68 has been shown to play a key regulatory role in the variable splicing process of the BCLx gene (Xu et al., 2022). The ZBTB7A protein can affect the variable splicing process of the BCLx gene by interacting with SAM68, resulting in a decrease in the binding capacity between SAM68 and BCLx mRNA, which leads to the splicing process favoring the proximal 5’ splice site and ultimately manifests the generation of BCLXL with antiapoptotic functions (Maeda, 2016). In addition, ZBTB7A affects cell cycle progression by regulating the transcription of genes related to cell cycle proteins (cyclins), cyclin-dependent kinase (CDK) and its inhibitor (CDKI), and transcriptional repression of the expression of genes that promote cell cycle progression, which causes cells to stagnate at a specific stage and inhibits excessive proliferation (Yang et al., 2012; Di Palo et al., 2020).

ZBTB7A promotes lymphocyte differentiation by inhibiting Notch (Redondo Monte et al., 2020a), a key cell lineage regulator. Moreover, ZBTB7A promotes the proliferation and survival of B cells by inhibiting the ARF–Mdm2–p53 pathway (Maeda et al., 2007; Bohn et al., 2014). Carpenter (Carpenter et al., 2017) reported that ZBTB7A determines the differentiation of helper T cells 17 (Th17) and regulatory T cells (Tregs). ZBTB7A protein expression was detected by intracellular staining in CD4^+^ and CD8^+^ single-positive thymocytes as well as in T cells cultured *in vitro* (Twu and Teh, 2014). In the presence of ThPOK and ZBTB7A, developing thymocytes differentiate into the CD4 lineage. Initial CD4^+^ T cells can differentiate into Th cells, including Th17 cells and regulatory T (Treg) cells (Zeidan et al., 2019). In addition, ZBTB7A inhibits the proliferation of adipocytes on the one hand by inhibiting the binding of the transcriptional activator Sp1 to the promoter region of the Cyclin A gene and, on the other hand, indirectly regulates the expression of peroxisome proliferator-activated receptor γ (PPARγ) by decreasing the expression of E2F-4, thereby promoting the terminal differentiation of preadipocytes (Laudes et al., 2004; Laudes et al., 2008).

#### 2.2.2 Tumor regulation

Initial studies suggested that ZBTB7A is a proto-oncogene (Lunardi et al., 2013); however, further studies have revealed that its role in cancer is environmentally dependent and that whether it is oncogenic or oncostatic depends on the specificity of the particular type of cancer and stage of the disease, as well as on molecular interactions. Researchers (Constantinou et al., 2019; Singh et al., 2021) have analyzed the link between ZBTB7A regulatory pathways and tumors using gene networks in Network Analyst, which revealed that ZBTB7A is involved in the regulation of pathways related to the TGF-β, ARF-MDM2-p53, NF-κB, PI3K/AKT, AR, AMP, NOTCH, FOXO and WNT signaling pathways. Comprehensive studies (Constantinou et al., 2019) have shown that ZBTB7A plays a central role in the formation of a wide range of tumors by regulating the P53, TGF-β and NF-κB signaling pathways. It involves functions such as epithelial mesenchymal transition, immune modification, alteration of glycolytic processes and non-coding RNAs (Tian et al., 2010; Constantinou et al., 2019). Survival analyses for breast, prostate, pancreatic, gastric, hepatocellular and leukemia cancers have shown that high expression levels of ZBTB7A correlate with poorer survival in these patients (Tian et al., 2010; Guo et al., 2014). Researchers (Guo et al., 2014; Li et al., 2015) are actively exploring ZBTB7A as a new target for the treatment of hepatocellular carcinoma, lung cancer and cervical cancer. However, ZBTB7A has bidirectional effects on tumorigenesis and suppression. In specific types of malignancies, such as early androgen receptor (AR)-driven prostate cancer and chronic lymphocytic leukemia, ZBTB7A inhibits tumor progression (Lee et al., 2002). In melanoma, ZBTB7A inhibits melanoma metastasis by transcriptionally repressing the expression of melanoma cell adhesion molecule (MCAM), which binds directly to the promoter region of MCAM and inhibits its expression, thereby reducing the invasive and metastatic capacity of melanoma cells (Liu et al., 2015). In addition, ZBTB7A can inhibit tumor metastasis by downregulating the expression of E-cadherin and β-catenin and inhibiting the expression of matrix metalloproteinases, which reduces the migratory and invasive ability of tumor cells (Guo et al., 2014; Mak et al., 2015).

#### 2.2.3 Metabolic regulation

Currently, ZBTB7A is considered a transcriptional repressor of genes encoding key enzymes or proteins involved in the glycolytic process (Liu et al., 2014). ZBTB7A directly binds to the promoter regions of GLUT-3, PFKP, and PKM and inhibits the transcription of these genes (Liu et al., 2014). GLUT-3 (Hochrein et al., 2022) is responsible for glucose transport into the cell, and PFKP Yang et al., 2023 and PKM (Xie et al., 2016) control key steps in the glycolytic process. By inhibiting the expression of glycolysis-related enzymes and limiting the rate of glycolysis, ZBTB7A can restrict the sugar metabolism of cancer cells and thus inhibit their proliferation. ZBTB7A deficiency leads to rapid tumor progression and is not useful for therapeutic drugs that target glycolysis, such as 2-deoxy-D-glucose (2-DG), which also reflects the importance of ZBTB7A in inhibiting the metabolism of tumor cells (Redondo Monte et al., 2020b). The metabolic regulatory role of ZBTB7A does not exist in isolation; ZBTB7A also interacts with other metabolic regulatory factors or signaling pathways to regulate cell metabolism. Under hypoxia, NF-κB is activated and undergoes nuclear translocation, where it binds to the NF-κB response element in the 5’ upstream regulatory region of the ZBTB7A gene and inhibits the expression of ZBTB7A, thereby increasing the transcription of MCT-4 and thus affecting the efflux of lactate, which is essential for the growth and survival of colon cancer cells under hypoxia (Choi et al., 2019). Kambe (Kambe et al., 2024) reported that the expression of ZBTB7A may be affected by hypoxia-inducible cellular metabolism; specifically, it may be regulated by hypoxia inducible factor-1 (HIF-1α), which accumulates in hypoxia and binds to chaperones (e.g., aryl hydrocarbon receptor nuclear translocator (ARNT)) to regulate the expression of target genes. In addition, under hypoxia, ZBTB7A may be involved in cell metabolism and angiogenesis by regulating related genes (e.g., vascular endothelial growth factor (VEGF)) (Leng et al., 2019). Therefore, ZBTB7A plays an important role in the regulation of cellular metabolism, especially glycolysis.

### 2.3 Distribution and expression regulation of ZBTB7A in the CNS

Studies have shown that ZBTB7A is expressed in the CNS, mainly in the cerebral cortex (prefrontal cortex), cerebellum, brainstem, and spinal cord (Fulton et al., 2023). ZBTB7A is distributed in a wide range of neuronal cells. In oligodendrocytes, its expression level is very low in oligodendrocyte precursor cells, whereas during myelin regeneration, its expression level increases mainly in mature oligodendrocytes (Dobson et al., 2012; Davidson et al., 2017). These findings suggest that ZBTB7A has specific distribution characteristics in different developmental stages of oligodendrocytes, which may be related to the maturation and myelin regeneration of oligodendrocytes. These findings suggest that ZBTB7A plays an important role in neuronal maturation. It may promote neuronal maturation and differentiation by regulating the expression of genes related to neuronal maturation [e.g., neurofilament light polypeptide (NF-L)] (Okado, 2021; Kim H. B. et al., 2025). In addition, ZBTB7A has been suggested to be a key transcriptional regulator in responsive astrocytes, and Fulton’s study (Fulton et al., 2023) confirmed that the translation of *Zbtb7a* mRNA was significantly upregulated in the cortical astrocytes of stress-susceptible mice, with no significant differences in the hippocampus or striatum, suggesting that *Zbtb7a* expression in astrocytes may be associated with the behavioral stress response. Currently, few studies have investigated the role of ZBTB7A in the CNS. However, ZBTB7A plays an important role in the regulation of glycolysis-based glucose metabolism, and astrocytes also play a key role in energy metabolism in the CNS. Therefore, ZBTB7A may play a regulatory role in astrocyte glycolysis.

## 3 The physiological basis of glycolysis in astrocytes

### 3.1 Energy metabolism in the CNS

The human brain is highly dependent on glucose uptake; although the brain is only approximately 2% of body mass, it consumes 20–25% of total glucose consumption (Magistretti and Allaman, 2015; Barros et al., 2018). The main substrates of brain tissue energy metabolism include glucose, lactate, and ketone bodies (Myette-Côté et al., 2022). The core of energy metabolism consists of two pathways, oxidative phosphorylation (OXPHOS) and glycolysis, which produce the intracellular energy donor adenosine triphosphate (ATP) (Watts et al., 2018; Myette-Côté et al., 2022). The proportions of these two pathways in the energy supply are adjusted in response to changes in cellular physiological processes. In mammalian embryos and juveniles, glycolysis is the main mode of cellular energy production, whereas in adulthood, there is a gradual shift to the coexistence of oxidative phosphorylation and glycolysis, with OXPHOS eventually becoming dominant (Peters et al., 2022; Barros et al., 2023). In addition, another important pathway for glucose metabolism in the CNS, the pentose phosphate pathway (PPP) (TeSlaa et al., 2023), is divided into oxidative and non-oxidative phases. On the one hand, PPP provides NADPH to neuronal cells as a hydrogen donor to participate in a variety of biosynthetic reactions and antioxidant defenses and, on the other hand, provides key raw materials for nucleotide synthesis, such as ribulose-5-phosphate and related materials (Stincone et al., 2015). Notably, lactate released from skeletal muscle and ketone bodies produced by the liver are transformed into important energy substances for the brain when glucose is in short supply (Magistretti and Allaman, 2018). The conventional view is that lactate production is associated with muscle fatigue and is a byproduct of energy metabolism in the body (Cai et al., 2022; Liu et al., 2024). However, there is growing evidence that lactate plays a broad role as a substrate for energy metabolism in the CNS and that the CNS prefers to utilize lactate as an energy source rather than glucose (Lei et al., 2024; Sezer et al., 2024). In other words, When glucose and lactate coexist, neurons may, under certain conditions, preferentially utilize lactate as an energy substrate (Świątkiewicz et al., 2023). In addition, lactate is necessary for the formation of long-term memories, and in recent years, there has been much evidence that lactate functions as a signaling molecule in the brain that promotes the expression of genes associated with synaptic plasticity (Wang et al., 2019; Fernández-Moncada et al., 2024).

### 3.2 The biological process of glycolysis

Glycolysis is the process of breaking down glucose or glycogen into pyruvate, ATP and NADH (Fernie et al., 2004; Li et al., 2023). This process takes place in the cytoplasm without oxygen and consists of a total of 10 consecutive steps (Alberghina, 2023; Kooshan et al., 2024), all of which are catalyzed by the corresponding enzymes. Notably, three of these steps are irreversible and are catalyzed by three rate-limiting enzymes, namely, hexokinases (HKs), phosphofructokinases (PFKs), and pyruvate kinases (PKs). The main process involves the transport of glucose by GLUTs through the cell membrane and into the cytoplasmic matrix, phosphorylated by HKs and converted to G-6-P, which is not able to pass through the cell membrane; this product is then catalyzed by a series of enzymes, including PFKs and PKs, to pyruvate. In cells under hypoxic or anaerobic conditions or with special glycolytic requirements, glycolysis can convert pyruvate to lactate via lactate dehydrogenases (LDHs).

**FIGURE 2:**
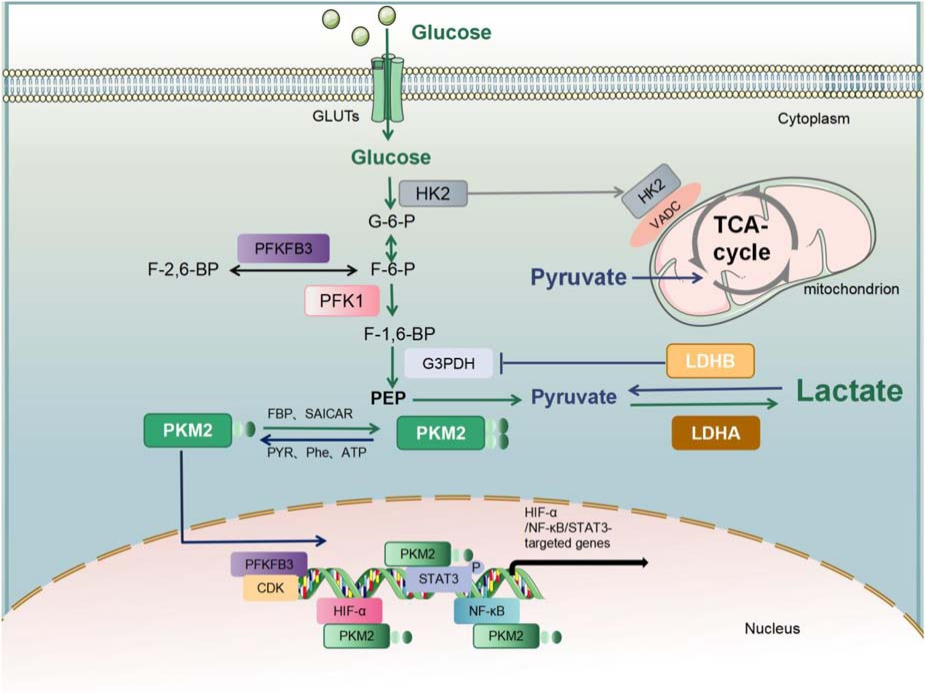
Biological processes, key enzymes and regulatory mechanisms of glycolysis. Glycolysis is the process of breaking down glucose or glycogen into pyruvate, ATP and NADH. This process is carried out in the cytoplasm and does not require oxygen In total, it consists of 10 consecutive steps, all catalyzed by the corresponding enzymes, and the three-step reaction catalyzed by three rate-limiting enzymes, HKs, PFKs, and PKs, is an irreversible reaction. The main process of glycolysis is that glucose is transported by GLUTs through the cell membrane into the cytoplasmic matrix, where it is converted to G-6-P, which is not able to pass through the cell membrane, through phosphorylation by HKs, and then catalyzed by a series of enzymes, such as PFKs and PKs, to pyruvate. Glycolysis can convert pyruvate to lactate via LDHs in cells under hypoxic or anaerobic conditions or special glycolytic requirements.

When a cell breaks down glucose in a specific way in the presence of sufficient oxygen, i.e., when the metabolic pathway changes from mitochondrial respiration to glycolysis and lactate production to meet its energy requirements, this process is known as aerobic glycolysis, which is also referred to as the non-oxidative metabolism of glucose or the “Warburg effect” (Vaupel and Multhoff, 2021; Zhong et al., 2022). In the aerobic glycolysis pathway, glucose is mainly converted to lactate, and this conversion is regulated by the glycolytic enzyme phosphoinositide-dependent kinase-1 (PDK1), which inhibits the activity of pyruvate dehydrogenase (PDH) through phosphorylation, thereby preventing pyruvate from being acetylated in the mitochondria (CoA) (Forteza et al., 2023). Pyruvate in the cytosol is subsequently converted to lactate via LDH and excreted via monocarboxylate transporter 1 (MCT-1) mediation. The high level of lactate accumulated by this process facilitates anabolic processes such as nucleotide synthesis (Imanaka et al., 2022; Forteza et al., 2023). Moreover, mitochondria-associated LDHB/C oxidizes lactate to pyruvate, thereby shifting cellular metabolism to a lactate-consuming mode. This mechanism allows the cell to efficiently recycle and utilize the lactate produced during its own metabolism as an additional source of energy. Through this oxidative recycling pathway, the cell not only reduces the direct consumption of glucose but also enhances the bioavailability of glucose, in line with the principle of optimization of energy metabolism. However, although aerobic glycolysis is much less efficient than mitochondrial OXPHOS in generating energy (aerobic glycolysis is 2 ATP/glucose, whereas mitochondrial OXPHOS can reach up to 36 ATP/glucose), it is able to rapidly provide ATP and substrates for biosynthesis pathways to satisfy the needs of rapid cell growth and proliferation (Kang et al., 2023). The “Warburg effect” is now considered a hallmark feature of not only tumor cells but also certain cells that are able to adjust their energy and substrate requirements in response to changing environmental conditions through aerobic glycolysis, which has been shown to be the only pathway of energy acquisition for mature erythrocytes, as well as for neurons, leukocytes, bone marrow and other cells under aerobic conditions (Kornberg et al., 2018; Schurr, 2018; Pan et al., 2022; Schurr and Passarella, 2022). The biological processes, key enzymes and regulatory mechanisms of glycolysis is shown in Figure 2.

### 3.3 Key enzymes in the glycolysis process

#### 3.3.1 HKs

HKs are the first key rate-limiting enzymes that regulate aerobic glycolysis and are responsible for converting glucose entering the cell into the membrane-impermeable glucose-6-phosphate (G-6-P) (De Jesus et al., 2022). Five hexokinase isoforms have been identified in mammals (Heneberg, 2019): HK1 is widely distributed in the brain (Okur et al., 2019; Zhang et al., 2020); HK2 is found mainly in the heart, muscle, adipose, and skeletal tissues (Tan and Miyamoto, 2015); and HK3 is widely distributed in the bone marrow, lungs, and spleen (Lin et al., 2018). GCK plays a key role in the regulation of pancreatic insulin secretion as well as hepatic uptake, synthesis, and catabolism during gluconeogenesis (Xu and Herschman, 2019). HKDC1 is closely related to the human hexokinase gene and is genetically adjacent to HK1 (Danial et al., 2003; Shi et al., 2019). HK catalysis is characterized by a low affinity for glucose and is hormonally regulated, with long-chain fatty CoA and G-6-P inhibiting hexokinase activity and insulin activating hexokinase activity (Aguilera-Alvarado and Sánchez-Nieto, 2017; Heneberg, 2019). Currently, HK-2 is the most widely studied protein in the clinic. HK2 has a dual role in tumor cells (Tan and Miyamoto, 2015). On the one hand, it controls glycolysis flow (Chen et al., 2023; Rho et al., 2023); on the other hand, it inhibits apoptosis by binding to voltage-dependent anion channels (VDACs) in the outer membranes of mitochondria (Ciscato et al., 2021; Xie et al., 2024).

#### 3.3.2 PFKs

G-6-P is catalyzed by PFK1 to generate fructose-1,6-bisphosphate, the second irreversible rate-limiting step of the glycolytic process (Al Hasawi et al., 2014). There are three isoforms of PFKs: platelet-type (PFKP), muscle-type (PFKM) and liver-type (PFKL) (Campos and Albrecht, 2023). These three isoforms of phosphofructokinase are expressed in the brain and a variety of other tissues (Zuo et al., 2021). PFK1 fully regulates the flow of the glycolytic pathway. PFK1 catalyses the conversion of fructose 6-phosphate to fructose 1,6-bisphosphate and adenosine diphosphate (ADP) with ATP, thereby driving glycolysis (Campos and Albrecht, 2023). Its activity is metabolically regulated by a variety of metabolites, with ATP, citric acid, and phosphoenolpyruvic acid having inhibitory effects and high concentrations of fructose 2,6-bisphosphate, ADP, and adenosine phosphate having activating effects (Mor et al., 2011). In addition, phos-phofructokinase-2/fructose-2,6-bisphosphatase 3 (PFKFB3) does not act as a rate-limiting enzyme in the regulation of the glycolytic pathway but rather catalyzes the production of fructose-2,6-bisphosphate, which can act as a metastable activator of PFK1 and significantly increase the catalytic activity of PFK1 (Kotowski et al., 2021). PFKFB3 regulates cyclin-dependent kinase (CDK) activity by translocating into the nucleus, leading to cell cycle arrest and the inhibition of cell death (Harold et al., 2023).

#### 3.3.3 PKs

The PKLR and PK genes encode four isozymes (Israelsen and Vander Heiden, 2015). PKLR encodes the L- and R-type proteins, which are expressed in erythrocytes and the liver, respectively (Luke et al., 2023). PK encodes the M1- and M2-type proteins, which are dependent on alternate splicing of the pre-mRNA, where the M1-type protein encodes only exon 9, which is primarily responsible for catalyzing oxidative phosphorylation in organisms, and the M2-type protein encodes only exon 10, which catalyzes the process of aerobic glycolysis (Toller-Kawahisa et al., 2023). PKM2 catalyzes the production of pyruvate and ATP by transferring a phosphate group from phosphoenolpyruvic acid (PEP) to ADP (Toller-Kawahisa et al., 2023). Heterogeneous nuclear ribonucleoprotein A1 (hnRNPA1) (David et al., 2010), hnRNPA2 (David et al., 2010) and polypyrimidine bundles (PTBs) (Huang et al., 2023) specifically bind to introns on both sides of exon 9 (E9) in PKM pre-mRNA, inhibit the shearing process of E9, promote the expression of E10, and thus increase the formation of exon 10-containing PKM2. The PKM2 tetrameric state has pyruvate kinase activity, and the dimer can translocate to the nucleus to act as a coactivator of several transcription factors, such as HIF-1α (Wang et al., 2014; Zhang Y. et al., 2024), NF-κB (Wang et al., 2024), and signal transducer and activator of transcription 3 (STAT3) (Pucino et al., 2019; Shen et al., 2023). The switch between the PKM2 dimer and tetramer is regulated by endogenous and exogenous activators and inhibitors of transmutation regulation (Anastasiou et al., 2012; Zhang et al., 2019). In normally proliferating cells, when fructose 1,6-bisphosphate (FBP) reaches a certain level, it stimulates the conversion of PKM2 dimers to tetramers, which contributes to the glycolytic process (Zhang et al., 2019). However, when PKM2 binds to tyrosine-phosphorylated peptides, FBP is released, and its pyruvate kinase activity is inhibited. This regulatory mechanism enables the metabolites of glucose to shift from the energy-producing pathway to the anabolic pathway to meet the demands of cell growth and proliferation (Wu et al., 2024).

#### 3.3.4 LDHs

LDH belongs to the 2-hydroxy acid oxidoreductase family, and its core function is to catalyze the reversible conversion between pyruvate and lactate (Robin et al., 2023). In mammals, there are several isoenzymes of LDH, namely, LDHA, LDHB, LDHC, and LDHD (Chatterjee et al., 2023). LDHA functions as a “molecular switch” for the transition from glycolysis to oxidative phosphorylation in the glucose metabolic pathway and facilitates the conversion of pyruvate to lactate (Chen et al., 2022; Sharma et al., 2022; Zhu et al., 2023). LDHB is the core enzyme of the lactate oxidation pathway and is responsible for catalyzing the reaction between lactate and NAD^+^ to produce pyruvate, NADH and H+ (Fan et al., 2021). LDHB is involved not only in influencing vesicle maturation but also in regulating the acidification state of lysosomes and influencing the maturation of vesicles (Zhang P. et al., 2024). An NAD^+^-related glycolytic enzyme, glyceraldehyde-3-phosphate dehydrogenase (G3PDH) (Bal-Klara, 1989), has a competitive inhibitory relationship with LDHB, as well as a metabolic inhibitory effect of lactate on HKs and PFKs, a process that negatively regulates glycolytic processes (Zhang P. et al., 2024). LDHC is present specifically in the testes and spermatozoa of mammals and birds and is a key enzyme in sperm energy metabolism (Goldberg et al., 2010). Lactate and pyruvate are preferentially used as energy substrates over glucose in male germ cells after meiosis (Odet et al., 2008). Compared with LDHA/B, LDHC has greater thermal stability and broader substrate adaptation; however, the specific mechanism of LDHC action is still under intensive study.

### 3.4 Characteristics and function of glycolysis in astrocytes

**FIGURE 3:**
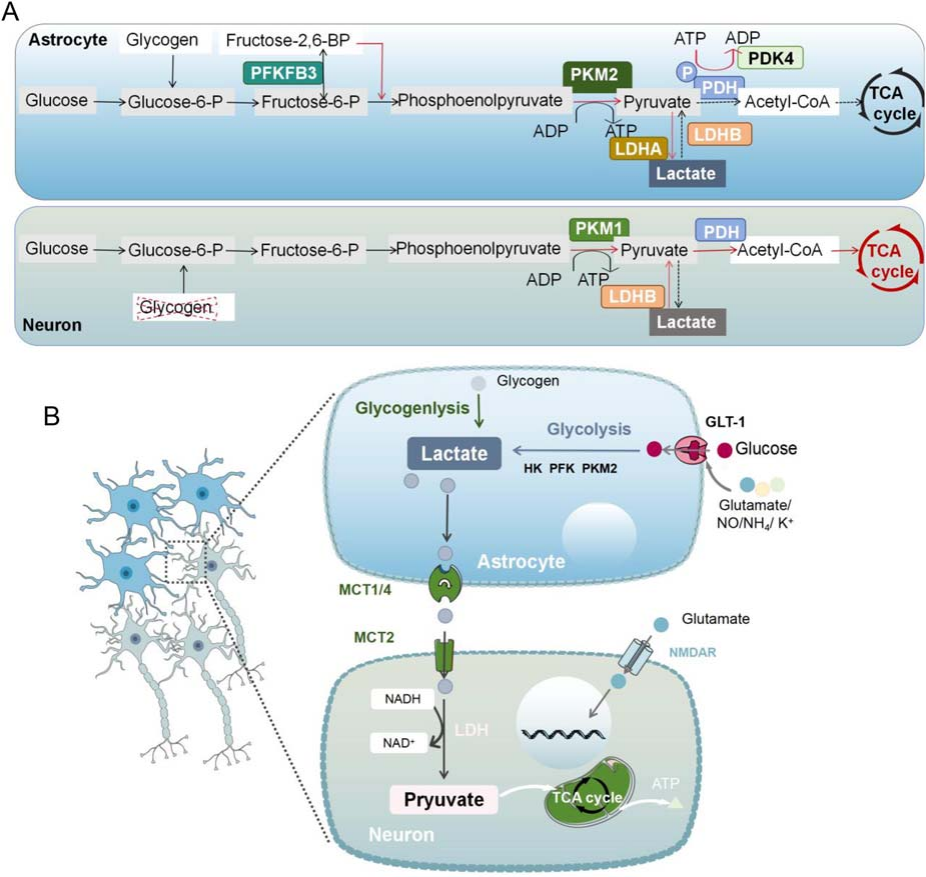
**(A)** Differential expression of energy metabolizing enzymes in astrocytes, neurons. Pyruvate kinase family: in astrocytes, PFKFB3 (a key positive regulator of glycolysis) and PKM2 (glycolysis-promoting) are highly expressed, promoting pyruvate production; in neurons, only PKM1 is expressed, suggesting low glycolytic activity in neurons. Pyruvate dehydrogenase family: in astrocytes, PDK4 is highly expressed and phosphorylates PDH, with a low flow of pyruvate into the TCA cycle; in neurons, PDH activity is high, which favors the entry of pyruvate into the TCA cycle, suggesting a high oxidative phosphorylation activity in neurons. Lactate dehydrogenase family: astrocytes predominantly express LDHA (the form of LDH that drives glycolysis); neurons predominantly express LDHB, which catalyzes the conversion of lactate to pyruvate. **(B)** Astrocyte-neuron lactate shuttling. Glucose from the circulation enters astrocytes via GLT1 and undergoes a series of reactions to generate lactate and release it into the extracellular space by MCT1 or MCT4 in the presence of a series of enzymes including HK, PFK and PKM2. Subsequently, extracellular lactate is taken up by MCT2 on the surface of the neuron and converted to pyruvate in the presence of LDH to complete the energy supply within the neuron.

Astrocytes are a key part of the “tripartite synapse” (astrocytes, presynaptic neurons, and postsynaptic neurons) in the CNS and are responsible for neuronal support, compartmentalization, nutrition and metabolism (Zhou et al., 2019; Endo et al., 2022). In the CNS, astrocytes deliver nutrients from the bloodstream to neurons and, in addition, store them in the endoplasmic reticulum in the form of glycogen or as free glucose, providing fuel reserves for neurons (Stogsdill et al., 2017). Astrocytes and neurons have different metabolic profiles (Lovatt et al., 2007; Vilchez et al., 2007; Figure 3A). For example, PKM1 and PKM2 are expressed in a cell-specific manner due to the selective shearing of PKM pre-mRNA, and PKM2 is highly expressed during the proliferation of embryonic neurons; however, as neurons differentiate, PKM2 levels decrease, and PKM1 levels increase. In contrast to neurons, differentiated astrocytes consistently express high levels of PKM2 (Bélanger et al., 2011; Falkowska et al., 2015). In addition, in astrocytes, PDK4 is highly expressed and phosphorylates PDH, and pyruvate enters the tricarboxylic acid (TCA) cycle at a lower level of flux. In neurons, PDH activity is increased, favoring pyruvate entry into the TCA cycle, indicating high oxidative phosphorylation activity in neurons. Whereas LDHA (the form of LDH that drives glycolysis) is expressed predominantly in astrocytes, neurons predominantly express LDHB, which catalyzes the conversion of lactate to pyruvate (Falkowska et al., 2015; Cunnane et al., 2020). The differential cellular expression of these enzymes is responsible for the preferential uptake of glucose from the blood by astrocytes, which produce lactate via the glycolytic pathway as the initiating stage of ANLS (Bélanger et al., 2011). The generated lactate is expelled from the extracellular space via MCT1 and MCT4. In the extracellular space, lactate is recognized by MCT2 on the neuronal membrane and transported into the neuron, i.e., completion of ANLS (Bélanger et al., 2011; Nortley and Attwell, 2017; Kim Y. et al., 2025; Figure 3B).

Glutamate is transported into the cell via Na^+^-dependent glutamate transporter-1 (GLT-1) and glutamate aspartate transporter (GLAST) to drive the astrocyte glycolytic pathway, increasing glucose utilization and lactate flux (Choi et al., 2012; Falkowska et al., 2015). NH4^+^, K^+,^ and NO all contribute to astrocyte glycolysis (Choi et al., 2012; Xu et al., 2013; Saez et al., 2014; Falkowska et al., 2015). NH4^+^ enters the mitochondria through K^+^ channels and transporters, leading to acidification of the mitochondrial matrix and a decrease in the pH gradient, which in turn inhibits the activity of the mitochondrial pyruvate carrier (MPC), leading to a shift of pyruvate to the lactate pathway, which provides energy to neurons. Astrocytes, as the only glycogen storage cells in the brain, play a key role in glycogen metabolism to maintain neuronal activation and glutamatergic neurotransmission (Brown, 2004; Lerchundi et al., 2015). G-6-P synthesizes glycogen catalyzed by glycogen synthase (GS), which is catalyzed by glycogen phosphorylase to produce pyruvate, which is converted to lactate for release into the extracellular compartment (Falkowska et al., 2015). Through transcriptomic sequencing, researchers have shown that since the rate-limiting enzyme of the pentose phosphate pathway, glucose 6-phosphate dehydrogenase, is highly expressed in astrocytes, it facilitates the generation of reducing-equivalent NADPH, which provides electrons for glutathione (GSH) production, as well as the maintenance of redox homeostasis (Brekke et al., 2017). Thus, there is a tight energetic interplay and metabolic synergy between astrocytes and neuronal activity via the glycolytic pathway.

## 4 The role of altered glycolysis in astrocytes in neurodegenerative diseases

### 4.1 The role of altered glycolysis in neurodegenerative diseases

Neurons, as the most energy-consuming cell type in the body, show a high degree of sensitivity to any subtle abnormalities in energy metabolism (Minhas et al., 2024). Defects in CNS energy metabolism that are closely associated with synaptic dysfunction have been observed in the early stages of AD (Minhas et al., 2024). Glycolysis is a major metabolic pathway whose decreased flux is closely associated with Aβ deposition, tau protein hyperphosphorylation and apoptosis (Butterfield and Halliwell, 2019). Brain glycolysis levels have been shown to be significantly decreased in significant regions of Aβ deposition (Finsterwald et al., 2021). Further experiments revealed that when neuronal cells are exposed to the glycolysis inhibitor ganglioside, the production of hyperglycosylated end products is promoted, and these products in turn promote tau protein hyperphosphorylation and apoptosis (Finsterwald et al., 2021). In addition, during normal aging, brain glycolysis levels also tend to decrease, which results in a deficiency in cerebral neurotrophic nutrition, prompting the brain to adapt by decreasing synaptic density, which may be one of the reasons for the gradual decline in cognitive function in elderly individuals, a process that may be related to the decrease in the amount of lactic acid required by neurons due to decreasing levels of glycolysis (Cunnane et al., 2020; Zheng et al., 2021). Notably, excessive enhancement of glycolysis is equally predictive of pathological changes in AD. In the brains of patients with AD, significant increases in the expression levels of glycolytic enzymes were observed with a decreasing trend in oxygen consumption, which directly led to increases in glucose uptake and lactate production (Bigl et al., 1999; Tang, 2020; Preeti et al., 2022). A significant shift in the pattern of glucose metabolism in the brains of patients with AD was revealed, from a dependence on mitochondrial respiration to a preference for glycolysis, which in turn resulted in a significant reduction in the efficiency of glucose utilization (Bigl et al., 1999). A moderate amount of lactate can effectively alleviate the problem of insufficient energy supply via transport to neurons as an energy source, promoting the expression of genes related to synaptic plasticity and increasing synaptic density, thus alleviating the symptoms of AD to a certain extent. However, when the level of lactate is too high, it not only inhibits the glycolysis process itself but also may disturb the acid–base balance of the body, triggering instability of the internal environment (Bigl et al., 1999; Yan et al., 2020). This disturbance of the internal environment may trigger a series of adverse reactions, including adverse effects on the nervous system. Exercise is widely recognized as having therapeutic benefits for PD and also promotes lactate production (Cai et al., 2019; Takahashi and Mashima, 2022). In neurons, lactate is an important energy substrate that compensates for ATP deficiency caused by α-synuclein toxicity, thereby improving the survival of dopaminergic neurons (Yang et al., 2024). Increased phosphoglycerate kinase 1 (PGK1) activity leads to increased production of pyruvate, a product of glycolysis. Terazosin (TZ) stimulates glycolysis by increasing PGK1 activity, increasing lactate levels in the brain, and ameliorating neuronal loss in an MPP-induced model of PD, resulting in elevated dopamine levels and partial recovery of motor function (Schultz et al., 2022). Thus, the use of lactate, a product of glycolysis, is expected to be a new way to treat PD.

### 4.2 The role of altered glycolysis in astrocytes in neurodegenerative diseases

Astrocytes, the most abundant cell type in the CNS and a major consumer of glucose, generate large amounts of lactate via aerobic glycolysis even in the presence of sufficient oxygen, which is subsequently transferred to neurons via ANLS as a substrate for the TCA cycle (Andersen et al., 2022). However, in neurodegenerative diseases closely related to aging, such as AD and PD, there is a gradual decline in glucose metabolism in the brain as a whole, accompanied by a decrease in the level of aerobic glycolysis in astrocytes. Astrocytes in the brains of patients with AD have lower glucose uptake capacity and metabolic activity than do those in the resting state (Andersen et al., 2021; Andersen et al., 2022). In patients with familial AD, the same insufficient lactate production in astrocytes occurs, disrupting ANLS and making astrocytes less capable of neuronal support (Andersen et al., 2021). Animal experiments and clinical reports have further revealed that early in neurodegenerative disease, neuronal mitochondrial function is impaired, and OXPHOS is increased as a compensatory mechanism, thereby competing with lactate produced by astrocytes as fuel for OXPHOS. This competition for energy substrates is detrimental to energy acquisition in healthy neurons, driving neurodegenerative and pathological states and contributing to the gradual evolution of the brain from a normal aging state to a neurodegenerative state (Zhang et al., 2023). IHC staining revealed that MCT1/2/4 expression was significantly reduced in both brain tissues of the APP/PS1 mouse model, suggesting that the ANLS mechanism might be inhibited and that the administration of FGF21 intervention significantly increased the expression of MCT1/2/4 and LDHA, which in turn increased ANLS ability and improved cognitive dysfunction in AD model mice (Zhang et al., 2018). In addition, impaired glycolysis affects the uptake and metabolism of glutamate by astrocytes (Sun et al., 2020). Astrocytes regulate the glutamate–glutamine cycle through GLT-1 and GLAST. This process is highly dependent on the sodium–potassium pump on the cytosolic membrane and is accompanied by a large consumption of ATP; thus, the mitochondria of astrocytes tend to be distributed in the vicinity of these transporters to support the process of glutamate metabolism through the metabolic pathway of rapid ATP generation. GLT-1 and GLAST are decreased in the brains of patients with AD, which results in a reduced uptake of glutamate by astrocytes and the development of excitotoxicity in neurons. Meanwhile, astrocytes predominantly express GLUT1 on their membrane surface, and the reduced expression of GLUT1 in the AD model also resulted in a reduced level of energy metabolism (Pajarillo et al., 2019).

Metabolic reprograming of striatal astrocytes also occurs in HD, where the striatum exhibits a microenvironment of reduced glucose levels, in which the metabolic pattern of astrocytes shifts from glycolysis to fatty acid oxidation or the use of endogenous, non-glycolytic metabolites as alternative fuels for energy supply (Khakh et al., 2017; Brandebura et al., 2023). This metabolic reprograming triggers an increase in liposomal oxidative processes accompanied by the accumulation of reactive oxygen species (ROS). The excessive accumulation of ROS ultimately produces neurotoxic effects, which in turn drive the progression of HD (Paryani et al., 2024). Xu et al. (2005) reported that cystatin C (Cys C) stimulates downstream neurons to increase redox reactions through the activation of astrocyte glycolysis, the release of lactic acid, and the increase in the capacity of neurons to eliminate hyperoxidative free radicals and glycolysis-based energy metabolism, thereby protecting dopaminergic neurons and delaying PD progression.

## 5 The role of ZBTB7A-regulated glycolysis in astrocytes in neurodegenerative diseases

### 5.1 Literature screening and database prediction of key genes of glycolysis regulated by ZBTB7A

ZBTB7A acts as a transcriptional activator or repressor mainly by specifically binding to DNA elements located on the DNA-binding domains of target genes and synergistically represses transcription through the recruitment of corepressor complexes (NCoR and HDAC) (Jeong et al., 2023). Many specialized transcription factor databases, such as AnimalTFDB, JASPAR, hTFtarget, and TRRUST, are available. These databases contain information on the sequences, structures, binding sites, and other features of known transcription factors. Predicting the binding sites of potential transcription factors to target genes by comparing the sequences to be predicted with the information in the databases or by analyzing the motifs in the DNA sequences is the first step in studying the functions of transcription factors. Glycolysis consists of a total of 10 consecutive steps (Fernie et al., 2004; Liu et al., 2014; Li et al., 2023), and each reaction step is essentially catalyzed by specific enzymes, including HKs, glucose phosphate isomerases (GPIs), PFKs, aldolases (ALDOs), triose phosphate isomerases (TPIs), glyceraldehyde-3-phosphate dehydrogenases (GAPDHs), PGKs, phosphoglycerate mutases (PGAMs), enolases (ENOs), and PKs. Subsequently, pyruvate can be converted to lactate by LDHs. In addition, in organisms, glucose is transported into the cell by GLUT-3 on the surface of the cell membrane, whereas lactate generated by glycolytic processes in astrocytes is transported out of the cell by MCT1 or MCT4. We searched domestic and international literature and found two studies that predicted and validated that ZBTB7A targets key genes involved in the inhibition of glycolysis. Dong et al. (2023) used the Human Transcript Factor Database to predict that ZBTB7A has potential binding sites in the HK2 promoter (−80∼-95 bp and -185∼-198 bp) and LDHA promoter (−53∼-66 bp and -225∼-239 bp) regions. In human glioblastoma cell lines (U87 and U251), ZBTB7A specifically binds to the *HK2* promoter (−80∼-95 bp) and the *LDHA* promoter (−53∼-66 bp), and the overexpression of ZBTB7A significantly inhibits the expression of the HK2 and LDHA proteins, reduces the capacity for cellular aerobic glycolysis and decreases lactate production and glucose consumption. Liu et al. (2014) identified the binding sites in a luciferase reporter assay and confirmed that ZBTB7A binds specifically to the *GLUT3* (+ 37∼ + 42 bp and -45∼-47 bp), *PFKP* (−90∼-95 bp), and *PKM* (−111∼-116 bp) promoters and affects glycolytic metabolic flux in HeLa cells.

The data in JASPAR CORE, the core data network of the JASPAR 2024 database,^1^ are collected from the literature, supported by experimental evidence, and manually checked, making it a non-redundant, high-quality transcription factor motif database Rauluseviciute et al. (2024). Therefore, we obtained the motif of the transcription factor ZBTB7A (MA0750.2-CCGGAAGTG-) from the JASPAR database and used the genes encoding key enzymes or proteins involved in the glycolysis process as the target genes (in addition to those that have been reported in the literature). The target gene was searched in the “gene” database of the NCBI website, the species was selected as *Homo sapiens*, and the + 100∼-1,000 bp region of the transcription start site (TSS) of the gene was selected from FASTA, which is the promoter region by default. The Relative Profile Score threshold was set to 80% to predict the binding site of the transcription factor ZBTB7A with the promoter region of the gene and analyze the position and score of the binding site. It is generally accepted that the higher the Score value is, the greater the likelihood of binding of the transcription factor to the target gene at that binding site. The Relative Score is usually between 0 and 1; the closer the value is to 1, the more likely it is to bind to a transcription factor, and a binding site with a Relative Score > 0.8 has a high degree of confidence. Strand denotes the specific DNA strand where the predicted transcription factor-binding site is located, “+” denotes the sense strand, and “−” denotes the antisense strand. We screened the loci with Score > 8 and Relative Score > 0.8 by individually predicting the target genes, the results of which are summarized in Supplementary Table S1. The transcription factor ZBTB7A was predicted to have potential binding sites with the promoter regions of the *GPI*, *ALDOA*, *TPI*, *PGK1*, *PGAM1*, *MCT-1*, and *MCT-4* genes, and no binding sites were predicted for *GAPDH* or *ENO1*.

**FIGURE 4:**
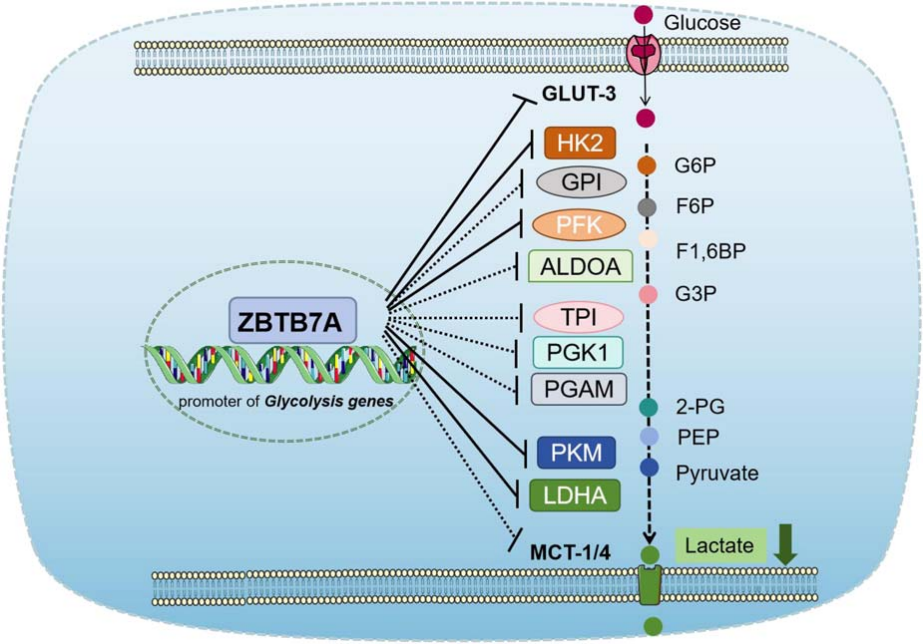
Literature screening and database prediction of key genes of glycolysis regulated by ZBTB7A. Literature screening: ZBTB7A binds specifically to the promoters of *HK2*, *LDHA*, *GLUT3*, *PFKP*, and *PKM* genes. Database prediction: The transcription factor ZBTB7A was predicted to have potential binding sites to the promoter regions of the *GPI*, *ALDOA*, *TPI*, *PGK1*, *PGAM1*, *MCT-1*, and *MCT-4* genes.

Therefore, combining domestic and international literature reports and predictions from transcription factor analyses through databases, it was concluded that ZBTB7A can bind to promoters of genes encoding key enzymes or proteins of the glycolytic process and that this binding is widespread in the glycolytic network, whereas its binding site is site specific (Figure 4). In biological processes, it can be assumed that ZBTB7A affects the glycolytic network and thus glycolytic metabolic flux by regulating the transcription of glycolytic genes. However, these bioinformatic predictions should be interpreted with caution. Motif-based scanning does not account for chromatin accessibility, histone modifications, transcription factor abundance, co-regulators, or cell-type-specific context, and therefore cannot establish actual binding in astrocytes. In particular, the predicted ZBTB7A-promoter interactions identified here have not yet been experimentally validated in astrocytes. Future studies will require experimental validations (e.g., gel mobility shift assay (EMSA) and chromatin immunoprecipitation sequencing (ChIP-seq)) luciferase reporter assays, and gain- or loss-of-function experiments *in vitro* and *in vivo*.

### 5.2 Potential mechanisms of ZBTB7A regulation of astrocytic glycolysis

Currently, studies on the regulation of astrocyte glycolysis by ZBTB7A have rarely been reported and are not yet fully understood. Through *in vitro* and *in vivo* experiments, researchers have demonstrated that transglutaminase 2 (TG2) in astrocytes shifts reactive astrocytes toward a phenotype that ameliorates the outcome of neuronal injury and that TG2 synergizes with ZBTB7A to repress transcription (Emerson et al., 2023). Using a mouse model of chronic stress exposure-induced major depression, researchers reported that ZBTB7A was significantly upregulated in orbitofrontal cortex (OFC) astrocytes but unchanged in neurons and microglia, suggesting that ZBTB7A is a key transcriptional regulator in reactive astrocytes (Fulton et al., 2023). Astrocytes generate and export lactate through aerobic glycolysis and ANLS, and MCT4 is a key transporter mediating lactate efflux from astrocytes. In addition, ZBTB7A has been reported to bind NF-κB response elements under hypoxic conditions and to regulate MCT4 transcription, thereby affecting lactate efflux (Choi et al., 2019) Although this mechanism was demonstrated in a non-astrocytic hypoxic context, it provides a plausible clue that ZBTB7A may also modulate astrocytic lactate handling through MCT4-related pathways.

It is important to note that most existing evidence for ZBTB7A as a glycolytic repressor comes from rapidly proliferating tumor cells (e.g., HeLa, U87, and liver cancer), where ZBTB7A suppresses glycolysis to limit anabolic metabolism and proliferation (Liu et al., 2014; Yang et al., 2016; Dong et al., 2023). By contrast, astrocytes are specialized for neuronal metabolic support through ANLS rather than self-replication, and their glycolytic program is characterized by high PKM2 expression, low PDH activity, prominent lactate production, and high MCT4 expression (Bélanger et al., 2011; Andersen et al., 2022). Therefore, if ZBTB7A represses glycolytic genes such as PKM, LDHA, or MCT4 in astrocytes, the outcome may differ from that in tumor cells: instead of primarily affecting proliferation, it could reduce lactate production and export, thereby disrupting ANLS and weakening neuronal energy support. Taken together, ZBTB7A may influence astrocytic glycolysis in a cell-context-dependent manner by modulating glycolytic flux and lactate transport. However, direct experimental evidence that ZBTB7A binds and functionally represses astrocyte-specific glycolytic genes remains limited, and this hypothesis requires further validation by *ex vivo* and *in vivo* studies.

### 5.3 ZBTB7A-regulated glycolysis in astrocytes in neurodegenerative diseases

We searched the literature with the keywords “ZBTB7A,” “astrocyte,” “glycolysis,” “neurodegenerative diseases” and were able to search relatively few related papers, but studies have shown that ZBTB7A plays an important role in other diseases related to the nervous system. ZBTB7A was found to be a transcriptional regulator of reactive astrocytes, and in the human OFC, it was shown to be a specific regulator of major depressive disorder (MDD) by transcription factor analysis and transcriptomics. ZBTB7A in astrocytes is involved in promoting chronic stress-induced behavioral deficits and neuronal hyperexcitability in the OFC (Fulton et al., 2023). Furthermore, in a study of GBM, ZBTB7A was found to be highly expressed in low-grade gliomas but significantly downregulated in patients with GBM. The mechanism involves ZBTB7A directly binding to the promoter of the bound *EPB41L5* gene and exerting a transcriptional repression function on the *EPB41L5* gene, thus inhibiting the progression of glioblastoma multiforme (GBM) (Jeong et al., 2023). Krossa et al. (2015) reported that ZBTB7A and Twist-1 coregulate apoptosis in glioblastoma cells. Knockdown of ZBTB7A resulted in increased caspase-3 or -7 activity and decreased chemotherapy resistance in glioblastoma cells, demonstrating that downregulation of ZBTB7A increases chemosensitivity in glioma. Yang et al. (2016) reported that resveratrol could effectively reduce the activity of the ZBTB7A promoter and its protein expression. In addition, resveratrol inhibited the DNA-binding activity of Sp1 and ZBTB7A, thus achieving antiglioblastoma proliferation efficacy. Moreover, ZBTB7A has a strong inhibitory effect on glycolytic genes, which is reflected in tumor cells; other studies have confirmed that ZBTB7A also plays a key role in the regulation of glycolysis in cardiomyocytes. Researchers overexpressed ZBTB7A in H9c2 cardiomyocytes by siRNA knockdown and lentiviral infection and reported that the silencing of ZBTB7A significantly upregulated the level of glycolysis in cardiomyocytes and that the overexpression of ZBTB7A reversed the increase in glycolysis induced by AngII (Cao, 2022).

**FIGURE 5:**
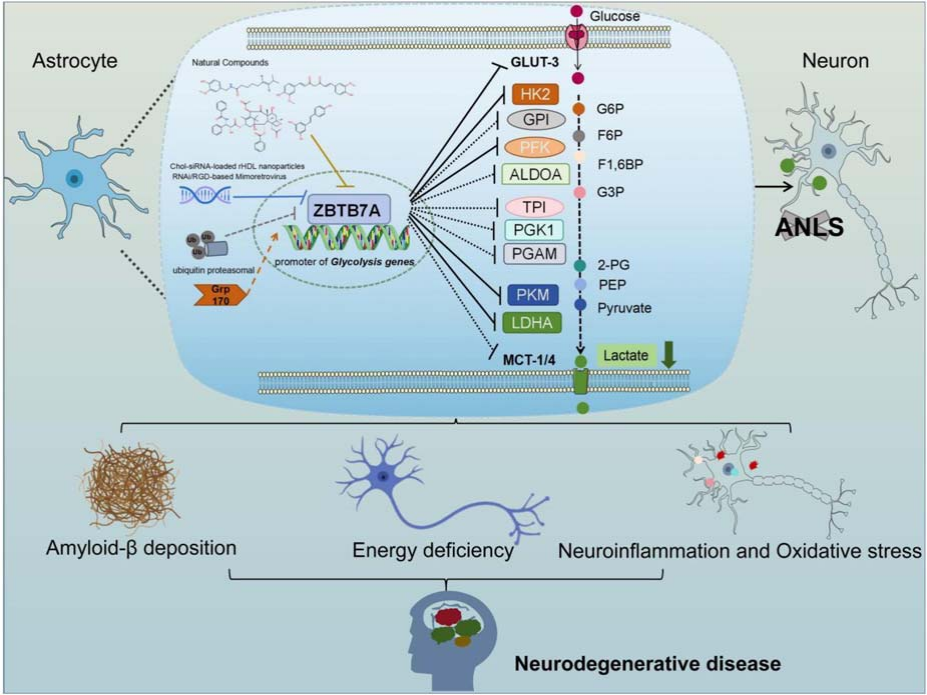
ZBTB7A-regulated glycolysis in astrocytes in neurodegenerative diseases. Combined with domestic and international literature reports and predictions through database analysis of transcription factors, ZBTB7A exerts transcriptional repression on glycolytic genes (Literature screening: *HK2*, *LDHA*, *GLUT3*, *PFKP* and *PKM*; Database prediction: *GPI*, *ALDOA*, *TPI*, *PGK1*, *PGAM1*, *MCT-1*, and *MCT-4*) in astrocytes, affects the glycolytic network, and reduces glycolytic metabolic flux, which not only affects ANLS and reduces neuronal energy supply, but may also affect the ability of astrocytes to clear Aβ, triggering neurotoxicity and further aggravating neuronal damage and Cognitive dysfunction. In addition, when ZBTB7A-regulated glycolysis is abnormal, insufficient supply of lactate may lead to disturbed energy metabolism in dopaminergic neurons, making them more sensitive to oxidative stress and inflammatory injury, and thus promoting dopaminergic neuron death. Strategies such as natural product therapy, gene silencing, and emerging degradation technologies based on protein interactions have the potential to target inhibition of ZBTB7A expression to intervene in a variety of disease processes.

Therefore, no study has yet clarified the role of ZBTB7A in neurodegenerative diseases such as AD and PD, given its important role in the regulation of astrocyte function as well as the regulation of gene expression associated with CNS diseases. This review combines the structure and function of ZBTB7A, as well as its distribution and expression regulation in the CNS, with the role of altered astrocyte glycolysis in neurodegenerative diseases, and it can be hypothesized that there is a potential association between altered astrocyte glycolysis regulated by ZBTB7A and neurodegenerative diseases. In the brains of patients with AD, the impaired glycolytic function of astrocytes leads to an insufficient energy supply, which is closely related to the pathological process of AD. ZBTB7A-regulated inhibition of astrocyte glycolysis may play an important role in this process. Combined with domestic and international literature reports and transcription factor analyses through databases, it is predicted that in biological processes, ZBTB7A exerts transcriptional repression on glycolytic genes (reported in the literature: *GLUT3*, *PFKP*, *PKM*, *HK2*, *LDHA*; database prediction: *GPI*, *ALDOA*, *TPI*, *PGK1*, *PGAM1*, *MCT-1*, *MCT-4*) in astrocytes, affects the glycolytic network, and reduces glycolytic metabolic fluxes, which not only affects the ANLS and reduces the energy supply of neurons but also may affect the ability of astrocytes to scavenge for Aβ, triggering neurotoxicity and further aggravating neuronal damage and cognitive dysfunction. In addition, ZBTB7A-mediated abnormalities in astrocyte glycolysis may affect the supportive function of ZBTB7A in dopaminergic neurons. Lactate produced by glycolysis is an important energy substrate for dopaminergic neurons. When ZBTB7A-regulated glycolysis is abnormal, an insufficient supply of lactate may lead to disruption of energy metabolism in dopaminergic neurons, increasing their sensitivity to oxidative stress and inflammatory injury and thus promoting dopaminergic neuron death (Figure 5).

## 6 Therapeutic agents targeting ZBTB7A

Currently, the development of ZBTB7A inhibitors is still at an early stage, and there is no search for clinically permissible therapeutic drugs approved by the U.S. Food and Drug Administration (FDA) that directly target ZBTB7A. We searched the national and international literature on the use of ZBTB7A for the treatment of related diseases and found that targeted inhibition of ZBTB7A expression through strategies such as natural product therapy, gene silencing, and emerging degradation technologies based on protein interactions has the potential to intervene in the progression of a variety of diseases (Table 1). Natural product compounds, which have a wide range of biological activities and the ability to act via multiple targets and signaling pathways, as well as good biocompatibility and relatively few side effects, have gradually demonstrated the irreplaceable properties of chemically synthesized drugs in the treatment of a wide range of diseases (Bui and Nguyen, 2017; Shen et al., 2024). Cui et al. (2010) and Yang et al. (2016) reported that curcumin and resveratrol could effectively reduce the activity of the ZBTB7A promoter and its protein expression and simultaneously inhibit the DNA-binding activity of Sp1 and ZBTB7A to achieve the antiproliferative efficacy of antitumor cells. Curcumin and paclitaxel can synergistically inhibit the expression of the NF-κB signaling pathway by downregulating ZBTB7A and exert synergistic antiproliferative and proapoptotic effects on triple-negative breast cancer cells (Mao et al., 2022). Capsaicin significantly inhibited ZBTB7A protein expression *in vitro* and *in vivo*, and the nuclear translocation of NF-κB, the target gene of ZBTB7A, was subsequently reduced by capsaicin treatment (Chen et al., 2021). ZBTB7A overexpression significantly attenuated capsaicin-induced anti-proliferative and pro-apoptotic effects. siRNA interference is an effective method of specific gene silencing and is widely used in scientific research to knock down ZBTB7A expression, but therapeutically, delivery systems such as nanoparticles or viral vectors are needed. Researchers (Tian et al., 2010) constructed a “mock retrovirus” using an arginine-glycine-aspartate (RGD) peptide ligand and a polylysine (K18) fusion peptide to package a recombinant retroviral plasmid expressing an siRNA against ZBTB7A. The resulting simian retroviruses formed stable nanoparticles that inhibited cell proliferation and invasion and promoted apoptosis. Ding et al. (2012) developed a safe and efficient biomimetic nanocarrier for the delivery of cholesterol-conjugated small interfering RNA (Chol-siRNA) to silence ZBTB7A and treat hepatocellular carcinoma (HCC). In addition, indirect pathways for therapy can also affect the binding activity of ZBTB7A to other proteins (e.g., binding chaperones) or modulate the level of binding between the two proteins. The stress protein grp170 exhibits molecular chaperone activity by binding to large protein substrates. Heat shock leads to the formation of a natural chaperone complex between grp170 and ZBTB7A, which triggers a T-cell response. Studies have shown that the injection of the grp170-ZBTB7A complex into mice with lung cancer significantly inhibits tumor growth and increases the lifespan of the mice (Yuan et al., 2012). Investigators have shown that the HSP90-dependent p53-KLHL20-ubiquitin proteasome pathway recruits and degrades ZBTB7A, which in turn regulates ZBTB7A expression (Choi et al., 2023). Therefore, based on the new technology of targeted protein degradation chimaeras (PROTAC), the ubiquitination and degradation of ZBTB7A in cells may be possible by designing a bifunctional molecule that binds ZBTB7A at one end and recruits E3 ubiquitin ligase at the other end.

**TABLE 1:** Therapeutic agents targeting ZBTB7A.

Treatment mode	Title	Experimental model	References
Natural compounds	Curcumin	Human lung carcinoma A549 cells; MDA-MB-231 mammary cancer cells	(Cui et al., 2010; Mao et al., 2022)
	Resveratro	Human glioma U87MG, T98G, and U251 cells	(Yang et al., 2016)
	Paclitaxel	MDA-MB-231 mammary cancer cells	(Mao et al., 2022)
	Capsaicin	Human breast cancer cell lines (MCF-7 and MDA-MB-231)	(Chen et al., 2021)
Genetic silence	RNAi/RGD-based Mimoretrovirus targeting ZBTB7A	SiHa cell	(Tian et al., 2010)
	Chol-siRNA-loaded rHDL nanoparticles targeting ZBTB7A	human hepatocellular carcinoma cell line HepG2	(Ding et al., 2012)
Protein-protein interaction	Grp170-ZBTB7A complex	C57BL/6 mice were challenged with subcutaneous injection of Lewis cancer cells	(Yuan et al., 2012)
	p53-KLHL20-ubiquitin proteasomal	Computer model simulation	(Choi et al., 2023)

Although ZBTB7A is a promising therapeutic target, its physiological roles in normal tissues suggest that systemic inhibition may lead to off-target effects. Therefore, CNS-specific, and ideally astrocyte-specific, intervention will be important for future translational development. Potential strategies may include blood-brain barrier (BBB)-penetrant small molecules, local administration approaches such as intrathecal or intracerebroventricular delivery, and cell type-specific delivery systems, including astrocyte-preferential vectors or targeted nanoparticles. However, the feasibility, safety, and durability of these approaches remain to be fully established.

## 7 Perspective

Overall, ZBTB7A may represent a promising regulatory node linking astrocyte glycolysis to neurodegenerative disease progression. However, no direct experimental evidence currently links ZBTB7A to AD or PD pathology, and this hypothesis remains to be tested. As the proposed ZBTB7A-glycolytic gene interactions are primarily based on bioinformatics prediction, further experimental validation is required. Future studies should employ astrocyte-specific systems, including ChIP, promoter reporter assays, and loss-/gain-of-function approaches, together with metabolic flux analysis, lactate assays, and neuron-astrocyte co-cultures. From a translational perspective, therapeutic development remains challenging due to the BBB, potential off-target effects, and the pleiotropic nature of ZBTB7A. Future strategies will require BBB-penetrant compounds, local administration, or astrocyte-preferential delivery platforms with rigorous safety evaluation.

To fill the evidence gap, we propose a concrete framework: (1) examine ZBTB7A expression in AD/PD postmortem brains; (2) generate astrocyte-specific Zbtb7a conditional knockout mice crossed with AD/PD models; (3) validate predicted ZBTB7A-glycolytic gene interactions using ChIP-seq, EMSA, and luciferase assays in astrocyte-specific systems. If these challenges can be overcome, ZBTB7A-directed intervention may provide a new avenue for restoring astrocytic metabolic support and slowing neurodegeneration.

## Footnotes

1
https://jaspar.elixir.no/
