# Supplementary material for: ZBTB7A-mediated regulation of astrocytic glycolysis in neurodegenerative diseases: insights from literature review and bioinformatics prediction
Source: Front Aging Neurosci. 2026 Jun 24;18:1852019. doi: 10.3389/fnagi.2026.1852019 (PMC13342185; doi:10.3389/fnagi.2026.1852019)
Supplement: Supplementary file 1 [file Data_Sheet_1.docx]

**Supplementary tables：**

**Table S1：Prediction of the binding site of ZBTB7A to the promoter of glycolytic genes**

| **Transcription Factor** | **Target Gene** | **Score** | **Relative score** | **Start** | **End** | **Strand** | **Predicted sequence** |
| --- | --- | --- | --- | --- | --- | --- | --- |
| MA0750.2.  ZBTB7A | *GPI* | 10.514212 | 0.8714312 | 713 | 725 | + | ACCCGGACGTGCT |
|  |  | 8.48005 | 0.83250844 | 906 | 918 | + | CCCCGGAACAGCT |
|  | *ALDOA* | 10.864439 | 0.8781326 | 688 | 700 | + | AACTGGAAGTGGC |
|  | *TPI* | 9.738652 | 0.8565912 | 796 | 808 | - | GGCCAGAAGTGGT |
|  |  | 9.45626 | 0.85118777 | 515 | 527 | - | GGCAGGAACTGCA |
|  | *PGK1* | 11.700004 | 0.89412075 | 741 | 753 | - | AGCAGGAAGCGTC |
|  |  | 10.762336 | 0.8761789 | 932 | 944 | + | GCCGGGAAGGGGC |
|  |  | 10.087121 | 0.86325896 | 793 | 805 | + | TGCCGGACGTGAC |
|  |  | 8.505146 | 0.8329887 | 806 | 818 | + | AAACGGAAGCCGC |
|  |  | 8.396668 | 0.830913 | 634 | 646 | - | TCCCGGAACCACG |
|  | *PGAM1* | 10.81779 | 0.87724 | 245 | 257 | - | TTCAGGAAGGGGC |
|  |  | 10.296038 | 0.8672566 | 486 | 498 | - | ACCTGGAAGTGCT |
|  | *MCT1* | 10.430554 | 0.8698304 | 569 | 581 | - | AGCGGGAAGCGAC |
|  |  | 8.305978 | 0.8291777 | 414 | 426 | - | CTCGGGAAGTTCC |
|  | *MCT4* | 10.087751 | 0.86327106 | 689 | 701 | + | CCCAGGAAATGCC |
|  |  | 9.248477 | 0.84721196 | 963 | 975 | + | CTCAGGAAGTAAA |

**Table S2：Abbreviations**

| AD | Alzheimer's disease |
| --- | --- |
| ADP | adenosine diphosphate |
| ANLS | astrocyte-neuron lactate shuttle |
| APP | amyloid precursor protein |
| ALDOs | aldolases |
| AR | androgen receptor |
| ARNT | aryl hydrocarbon receptor nuclear translocator |
| ATP | adenosine triphosphate |
| Aβ | β-amyloid |
| BBB | blood-brain barrier |
| CDK | cyclin-dependent kinase |
| CDKI | cyclin-dependent kinase inhibitor |
| ChIP-seq | chromatin immunoprecipitation sequencing |
| CNS | central nervous system |
| CoA | coenzyme A |
| Cys C | cystatin C |
| 2-DG | 2-deoxy-D-glucose |
| EMSA | electrophoretic mobility shift assay |
| ENO | enolases |
| E9 | exon 9 |
| FBP | fructose-1,6-Bisphosphate |
| FDA | Food and Drug Administration |
| GAPDHs | glyceraldehyde-3-phosphate dehydrogenases |
| GBM | glioblastoma multiforme |
| GLAST | glutamate aspartate transporter |
| GLT-1 | glyceraldehyde-3-phosphate dehydrogenase |
| GLUT-3 | glucose receptor-3 |
| GPIs | glucose phosphate isomerases |
| GS | glycogen synthase |
| GSH | glutathione |
| G3PDH | glyceraldehyde-3-phosphate dehydrogenase |
| G-6-P | glucose-6-phosphate |
| hnRNPA1 | heterogeneous nuclear ribonucleoprotein A1 |
| HATs | histone acetyltransferases |
| HCC | hepatocellular carcinoma |
| HD | Huntington's disease |
| HDAC | histone deacetylase |
| HIF-1α | hypoxia-inducible factor 1α |
| HKs | hexokinases |
| LDHs | lactate dehydrogenases |
| LPS | lipopolysaccharide |
| MCAM | melanoma cell adhesion molecule |
| MCT-1 | monocarboxylate transporter 1 |
| MDD | major depressive disorder |
| MPC | mitochondrial pyruvate carrier |
| NADH | nicotinamide adenine dinucleotide |
| NCoR | nuclear receptor corepressor |
| NF-L | neurofilament light polypeptide |
| NF-κB | nuclear factor nuclear factor-kappa B |
| NLS | nuclear localization signal |
| OXPHOS | oxidative phosphorylation |
| OFC | orbitofrontal cortex |
| PD | Parkinson's disease |
| PDH | pyruvate dehydrogenase |
| PDK1 | phosphoinositide-dependent kinase-1 |
| PEP | phosphoenolpyruvate |
| PFKL | liver-type phosphofructokinase |
| PFKM | muscle-type phosphofructokinase |
| PFKP | platelet-type phosphofructokinase |
| PFKFB3 | phos-phofructokinase-2/fructose-2,6-bisphosphatase 3 |
| PGAMs | phosphoglycerate mutases |
| PGK1 | phosphoglycerate kinase 1 |
| PKM | pyruvate kinase M |
| POK | Pokémon |
| PROTAC | protein degradation chimaeras |
| PPARγ | peroxisome proliferator-activated receptor γ |
| PPP | pentose phosphate pathway |
| PTB | polypyrimidine-tract binding |
| ROS | reactive oxygen species |
| STAT3 | signal transducer and activator of transcription 3 |
| TCA | tricarboxylic acid |
| TG2 | transglutaminase 2 |
| Th17 | T cells 17 |
| TPIs | triose phosphate isomerases |
| Tregs | regulatory T cells |
| TSS | transcription start site |
| TZ | Terazosin |
| VDACs | voltage-dependent anion channels |
| VEGF | vascular endothelial growth factor |
| ZBTB7A | Zinc Finger and BTB Domain Containing 7A |
